# Determining the impacts of hospital cost-sharing on the uninsured near-poor households in Vietnam

**DOI:** 10.1186/1475-9276-13-40

**Published:** 2014-05-17

**Authors:** Duong Anh Vuong, Steffen Flessa, Paul Marschall, Son Thai Ha, Khue Ngoc Luong, Reinhard Busse

**Affiliations:** 1 Department of Medical Service Administration, Vietnam Ministry of Health, Hanoi, Vietnam; 2 Department of Health Care Management, Berlin University of Technology, Berlin, Germany; 3 Department of Business Administration and Health Care Management, Ernst-Moritz-Arndt-University of Greifswald, Greifswald, Germany

**Keywords:** Cost-sharing, Hospital unit cost, User fee, Catastrophic health expenditure, Vietnam

## Abstract

**Objectives:**

The study objective was to identify the size of different hospital financing sources for different hospital services and their impact on the uninsured.

**Methods:**

A panel dataset of 84 public general hospitals (2005–2008) with cross-section data on hospital activity and hospital revenue was created and used to calculate unit costs of different hospital services by applying multiple regression models. The resulting risk of catastrophic health expenditure (CHE) was estimated based on official income statistics.

**Results:**

Average user fees (UF) for outpatient visits and inpatient bed days were US$4.13 and US$20.27, while actual full costs (AFC) were US$8.41 and US$36.66, respectively. These unit costs were 2.5 times higher in hospitals at the central versus the provincial level. UF for surgical inpatient bed days were 3.6 times that of non-surgical treatments (US$47.50 vs. 12.87) and AFC 5.0 times (US$101.72 vs. 20.08). UF accounted for 44.6%-77.9% of the AFC, the rest (22.1%-55.4%) was provided by direct government support (DGS). One surgical inpatient treatment at either central or provincial hospital level and one non-surgical inpatient treatment at central hospital level, immediately pushed uninsured near-poor households at risk of CHE.

**Conclusions:**

Around 45% of hospital AFC was paid by DGS, the larger rest by UF. UF have become a great financial burden on the uninsured near-poor households, who have to pay for these out-of-pocket and therefore may not utilize even necessary services. If the rate of DGS were reduced, this would have the effect of increasing UF, but the savings to Government could be spent on subsidizing insurance to ensure that a larger part of the population can cover UF through insurance, especially the near-poor households.

## Introduction

The Socialist Republic of Vietnam is currently in the process of implementing major health care reforms. One major element of these reforms is a shift from a centrally planned system where health care services were provided to the population free of charge, to the decentralized and contracted social health insurance (SHI) model. The introduction of hospital cost-sharing under the mechanism of a fee-for-service scheme was started in 1989 [1,2], which aimed to improve financial capacity and sustainability of these health care institutions resulting in higher quality and reliability of care. There have been major achievements, such as new health technology development, better health care service provision, increasing financial support for hospital performance and relief of the financial burden on the government. However, in the process of ongoing reform, a mix of payments for health care services consisting of contributions from the state budget and “user fees”, which are either covered by SHI as third party payments (TPP) or out-of-pocket (OOP), developed, which caused a major controversy. Objections to the reform include (i) that it might reduce necessary utilization by the poor, who may not be able to afford the health care services; and (ii) that the role of the government in supporting population access to hospital services is unclear, since there is no clear policy to demarcate responsibility among the state, health insurance and service users [3-5]. The question remains of how much of the hospital service costs is now financed by user fees and what the impacts of this are on the service users, in particular those who are uninsured and have to pay by OOP. Does it lead to “catastrophic health expenditure” (CHE)? This question will be explored and answered in this paper by looking at the revenues and costs of two main hospital services, namely outpatient visits and inpatient stays for both surgical and non-surgical patients and for both central and provincial hospitals.

### Background

The current health care system was established in the northern part of Vietnam by the late 1950s, then in the south after reunification in 1975. The health care system was formed according to the four administrative levels of the state. These are, firstly, the central level, then the provinces, which are in turn divided into districts and communes. At the central level there are 41 hospitals (18 general and 23 specialized); at the provincial level there are 340 hospitals (124 general and 216 specialized); and there are 609 district general hospitals. One health commune station exists in each commune at the grassroots [6-8].

As in most countries in Central-Eastern Europe and Central Asia, after the collapse of the Soviet Union in the late 1980s, the country faced a socio-economic crisis due to a sudden cut of foreign aid, and free health care provision to the whole population was no longer available. The state budget is only sufficient to support for public health facilities in some main categories of salaries, administrative management, equipment, maintenance, consumables, and a small number of hospital fee exemptions for the very poor or vulnerable groups of patients [9-11]. The rest has been covered by the so called ‘User Fee’, which was introduced by the Vietnamese Government by the Ordinance of Private Medical and Pharmaceutical Practices and the Policy on Hospital Partial Fees. The user fee was first introduced in 1989 for inpatient services, with a partial hospital fee, then expanded to all in- and outpatient services. It allows hospitals to collect a fee, according to a fee-for-service (FFS) scheme, for certain services including consultation, drugs, consumables, blood infusions, diagnostic procedures, operative procedures, and hospital bed utilization [11]. The ranges of these services’ fee were issued by the Ministry of Health, with the basic threshold determined for each relevant administrative level. The local authorities take it as a basis to specify the precise fee for each service, in relation to the technical capacity of their hospital and their local community’s ability to pay [3,4,12],]. The hospitals at the central level normally receive more investment and are better equipped with technology; and being the highest level in the referral hierarchy, they logically receive patients with more severe illnesses. Consequently, the highest level of the central hospitals has a higher cost rate compared to provincial or district hospital levels for the same service [13].

In short, funding to hospitals is a combination of two main sources: state budget (bed-norm based provision) and user fees [12]. The state budget is, as in most developing and some industrialized countries, transferred to the hospitals in the form of line-item allocations from government health authorities, the rate of provision depending on the wealth of each province [14,15]; these allocations are meant to cover the fixed costs of the hospitals, especially for personnel and maintenance. There are two main sources of UF payments, which are meant to cover the variable costs: TPP and OOP payments. In 2007, TPP comprised 49% of population, which was made up by statutory health insurance for employees with 9% of the population, free health care for the poor (HCFP) with 18%, free health care for children under 6 years of age with 11%, and another 11% by voluntary health insurance [16]; OOP payments are made by those with no health insurance.

The free HCFP policy was started since following “Decision 139” of the Prime Minister on “health examination and treatment for the poor” in 2002 which provides free health insurance for the poor who were defined as those with a total income per year under 2,400,000VND in rural and 3,120,000VND in urban areas (by purchasing power parity [PPP] in 2002, equal to US$405; US$527, respectively) [17,18]. The HCFP was rather successful in achieving positive outcomes with a positive impact on increasing overall health care service utilization; reducing OOP expenditure for health care of the poor and the risk of catastrophic OOP spending [5,19]. However, aside from the defined poor households who were provided free health insurance, the near-poor households are now of the greatest concern for the government in regard to health insurance provision.

The near-poor households are defined as having an income between 201–260,000VND/head/month in rural and 261–338,000VND/head/month in urban areas; in the estimation of the average annual income of the near-poor by PPP, in 2008 it was equal to US$420 per head for both urban and rural groups [20]. The near-poor are roughly estimated to account for 14% of the population [21]. The risk of this group for CHE is now at the higher than for the poor; Nguyen and colleagues found that 24% of them have to borrow money to pay for outpatient treatment, compared to 20% of the poor and 12% of others [22]. The government made a policy to subsidize 50% of the health insurance premium for the near-poor households, but 90% of them are not yet covered [23].

## Methods

### Data set

As no patient-level data were available, we had to use aggregate statistics from hospitals, which formed the basis for our analysis. The data used in this study are thus facility-based data of annual statistical reports, extracted from the annual hospital statistical reports collected and administered by Ministry of Health (Department of Medical Service Administration), over 4 years (2005–2008). By regulation, every hospital has to annually submit the hospital statistical report to the Ministry of Health (by electronic mail or on paper). However, each year about 15-20% of the observations were not available. The missing reports were those sent by post where the address may have been incorrect or the data administrators were not able to manually enter all data into the database at the MOH. General hospitals at the central and provincial levels which have submitted a minimum of 4 year reports to the Ministry of Health were selected for this study. The set of available data included 84 general hospitals (76 provincial hospitals, 8 central hospitals) with a total of 336 observations. Private hospitals were excluded as our purpose is to establish the share of different financial sources for the public hospital unit costs.

A panel dataset was generated including out- and inpatient flows; treatment and care procedures; and hospital revenue from user fees and state budget. To balance the value of local currency (VND) by years and to be suitable for international benchmarking, hospital income figures were adjusted by PPP to US dollars (PPP in 2008: 7,688; 2007: 6,484; 2006: 6,158; 2005: 5,919) [17]. Unit costs of hospital services are defined in the current study as unit cost of outpatient visits and hospital stays (operationalized as inpatient bed days). The inpatient bed days were further categorized into surgical and non-surgical cases, depending on whether patients had received an operation or not [24,25]. A further division was done between hospitals at provincial and central level.

As the data provided only contained aggregate revenue received by the hospitals, we had to rely on the following assumption to calculate costs from revenue: Based on the fact that (a) all financial sources in the processing procedures that can contribute to final outputs are value-added [26] and (b) all public hospitals are non-profit organizations and must therefore balance revenue and costs, the revenue was substituted for costs in analyzing cost units. Costs were classified into three different categories: revenue from user fees (UF) as a proxy for variable costs, the state budget revenue as a proxy for fixed costs, especially maintenance and personnel, and actual full cost (AFC) as additionally including depreciation of capital.

The hospital revenue from UF revenue was available as being collected by TPP or OOP; and total revenue was equally available as the combination of UF revenue, bed-norm based provision of state budget, donation and others (generally called state budget). To calculate AFC, based on previous studies, we estimated that the annual depreciation rate of capital investment on equipment and buildings added 8.5% to total revenue [13,27,28]. The state budget plus the annual depreciation of capital investment (as these are only public hospitals) accounted for the direct share contributed by the government (the so-called direct government support (DGS)).

The three different categories of revenue, and respective costs, can be displayed by formulas as follows:

$$\begin{array}{l} \begin{array}{ll} \mathrm{UF}\_R\approx \sum & {}_{\mathrm{Variable}\;\mathrm{costs}}=\sum_{\mathrm{Cost}\;\mathrm{of}\;\mathrm{consultation},\mathrm{drug},\mathrm{consumable},}\\ & {}_{\mathrm{infusion},\mathrm{blood},\mathrm{diagnosed}\;\mathrm{test}\;\mathrm{procedures},\mathrm{operation}}\\ & {}_{\mathrm{procedures},\mathrm{hospital}\;\mathrm{bed}\;\mathrm{use}} \end{array}\\ \begin{array}{ll} \mathrm{Total}\_R=\mathrm{UF}\_R & +\mathrm{StateBudget}\_R\approx \sum_{\mathrm{Variable}\;\mathrm{costs}}+\sum_{\mathrm{Fixcosts}1}=\sum_{\mathrm{Variable}\;\mathrm{costs}}\\ & \;+\sum_{\mathrm{Cost}\;\mathrm{of}\;\mathrm{maintenance},\mathrm{salaries}/\mathrm{wages},\mathrm{management}/\mathrm{operation}} \end{array}\\ \begin{array}{ll} \mathrm{AFC}=\mathrm{Total}\_ & R\;x\;1.085\approx \sum_{\mathrm{Variable}\;\mathrm{costs}}+\sum_{\mathrm{Fixcosts}1}+\sum_{\mathrm{Fixcosts}2}=\sum_{\mathrm{Variable}\;\mathrm{costs}}\\ & \;+\sum_{\mathrm{Fixcosts}1}+\sum_{\mathrm{Cost}\;\mathrm{of}\;\mathrm{depreciation}\;\mathrm{on}\;\mathrm{equipments},\mathrm{depreciation}\;\mathrm{on}\;\mathrm{buildings}} \end{array} \end{array}$$

The results of UF for each unit of hospital services were judged in relation to the concept of households at risk of CHE, especially for those who have to pay for the hospital services OOP. The risk of CHE is immanent if OOP payments exceed 15-20% of a household’s annual income, depending on the threshold used [29-32]. The income was taken here as the average income per capita in 2008 (11,942,400VND) adjusted to US$ by PPP (equal to US$1,553). With the average number of persons in one household is 3.8 person [33] the average national household income was equal to US$5,901 and average near-poor household income was equal to US$1,596.

### Regression models

Using the equation of multiple regressions on hospital cost functions to calculate the final hospital unit costs:

$$R_{i,t}=b_{o}+\sum b_{i,t}X_{i,t}+e_{i}$$

In which, R_i,t_ stands for the revenue of hospital i at time t;

X_i,t_: X_1,t_ is predictor variable of inpatient bed day at time t and X_2,t_ is predictor variable of outpatient visit at time t;

b_0_: the plane’s reference position (intercept) defines the value of R when all X_i_ =0;

b_i_: a regression coefficient of the variable X_i_ on the total revenue R_i_ that quantify the effects of inpatient bed day (X_1_) and outpatient visit (X_2_) upon the hospital revenue R_i,_ respectively; e_i:_ error term [34,35].

### Statistical analysis

The linear regression on the longitudinal/panel data methodology was applied in STATA 10.0. Firstly, UF revenue was regressed (fixed-effect) on the output variables of inpatient bed days (InpBD) and outpatient visits (OutpV). Then, a similar regression was applied for total revenue (i.e. UF and state budget) in the relationship with InpBD and OutpV. Those two models resulted in the ratios of regression coefficients between InpBD and OutpV interactions on UF revenue and on total revenue respectively which suggests cost complementarities between InpBD, OutpV on UF revenue and those on total revenue. These ratios were in turn used to estimate the UF revenue/total revenue allocated relevant to the unit costs of InpBD and OutpV (Table 1).

**Table 1:** Results of the regression models

**Independent variables**	**Revenue through user fees (UF)**			** Total revenue (UF plus state budget) = direct provider cost**		
	**Coefficient**	**SE**	**t/P-value**	** Coefficient**	**SE**	**t/P-value**
	Dep Var: UF Revenue			Dep Var: Total Revenue		
	R-sq: within = 0.63			R-sq: within = 0.71		
**InpBD**	75.71	4.46	16.96/<.001	87.69	4.35	20.15/<.001
**OutpV**	15.42	4.26	3.61/<.001	20.15	4.16	4.84/<.001
	Dep Var: InpBD					
	R-sq: within = 0.76					
**SurInpC**	6.29	.624	10.07/<.001			
**OtherInpC**	7.21	.320	22.53/<.001			
	Dep Var: Cost of inpatient bed days			Dep Var: Cost of inpatient bed days		
	R-sq: within = 0.46			R-sq: within = 0.40		
**SurInpBD**	.0001646	.0000199	8.27/<.001	.0001859	.0000229	8.11/<.001
**OtherInpBD**	.0000446	.0000091	5.59/<.001	.0000367	.0000091	4.00/<.001

(*Abbreviations*: *InpBD* inpatient bed day, *OutpV* outpatient visit, *SurInpC* Surgical inpatient case, *OtherInpC* non-surgical inpatient case, *SurInpBD* Surgical inpatient bed day, *OtherInpBD* non-surgical inpatient bed day).

Similarly, the Regression Model (fixed-effect) was run for InpBD on two variables of surgical and non-surgical inpatient cases, to find the regression coefficient ratios of length of stay (LOS) for one surgical case (SurInpC) versus one non-surgical inpatient case (OtherInpC), that were used to calculate the number of surgical inpatient bed days (SurInpBD) and number of non-surgical bed days (OtherInpBD). Those two variables (SurInpBD, OtherInpBD) were then regressed on the UF revenue/total revenue to find the ratios of regression coefficients of one SurInpBD and one OtherInpBD interaction on the cost of inpatient bed days in regard to UF revenue and to total revenue, respectively. Then, relying on those regression coefficient ratios the total cost of surgical and non-surgical inpatient bed days were calculated (Table 1).

All fixed-effect (within) regression were tested by a Hausman Fixed Random test to make sure the difference in coefficients between fixed-effect (within) regression and random-effect GLS regression is not systematic [35,36].

## Results

From the baseline data:

The percentage of patients paying user fees through OOP was 46.7% on average for out- and inpatient visits; it declined from 56.2% in 2005 to 41.8% in 2008. Conversely, the percentage of patients covered by TPP increased from 43.8% to 58.2%. The LOS was almost stable, in the range of 7.3-7.6 days at the provincial hospital level and 9.6-10.4 days at the central hospital level (Table 2).

**Table 2:** Hospital characteristics by year

**Year**	**Average length of stay (in days)**			**% of patients for whom user fees are covered by TTP (n = 84)**
	**All (n = 84)**	**Province (n = 76)**	**Central (n = 8)**	
**2005**	7.7	7.4	10.4	43.8
**2006**	7.8	7.6	10.1	51.9
**2007**	7.7	7.5	10.0	57.2
**2008**	7.6	7.3	9.6	58.2

From the Regression Model:

A significant linear regression was found between UF revenue and two variables of InpBD and OutpV (both variables, p < .001) and accounted for 63% of the variance in UF revenue (R^2^ = .63). Similar observations were found for total revenue in the relationship with InpBD and OutpV (R^2^ = .71; both variables, p < .001). The ratio of regression coefficients between InpBD and OutpV interactions on UF revenue is 75.71/15.42 and on total revenue is 87.69/20.15 (Table 1).

Based on UF revenue, the variable costs of one InpBD and OutpV were US$20.27 and US$4.13, respectively; one SurInpBD cost US$47.50 versus US$12.87 for an OtherInpBD. Comparing unit costs between different hospital levels, the hospitals at the central level cost 2.5 times more than the ones at the provincial level (outpatient visit: US$9.22/3.59, inpatient bed day: US$45.28/17.64) (Table 3 ).

**Table 3:** Means of unit costs for hospital services and percentage of UF per AFC of each unit cost

**Hospital service and level**	**Mean of unit costs (US$)**			**UF as% of AFC**
	**By UF ≈ variable costs [95% CI]**	**By total revenue ≈ variable plus fixed costs [95% CI]**	**Actual full costs (AFC)**	
**Outpatient visit**				
All	4.13 [3.76-4.49]	7.76 [7.32-8.20]	8.41	49.10
Provincial level	3.59 [3.35-3.83]	7.04 [6.72-7.35]	7.63	47.05
Central level	9.22 [6.59-11.85]	14.64 [12.03-17.25]	15.88	58.06
**Inpatient bed day**				
All	20.27 [18.47-22.08]	33.79 [31.87-35.70]	36.66	55.29
Provincial level	17.64 [16.46-18.82]	30.63 [29.27-31.99]	33.23	53.08
Central level	45.28 [32.38-58.18]	63.74 [52.38-75.10]	69.15	65.48
**Surgical inpatient bed day**				
All	47.50 [43.59-51.40]	93.76 [88.93-98.58]	101.72	46.69
Provincial level	41.70 [39.25-44.16]	86.10 [82.73-89.47]	93.41	44.64
Central level	102.53 [74.08-130.97]	166.46 [136.03-196.89]	180.60	56.77
**Non**-**surgical inpatient bed day**				
All	12.87 [11.81-13.92]	18.51 [17.55-19.46]	20.08	64.09
Provincial level	11.30 [10.63-11.96]	16.99 [16.33-17.66]	18.43	61.31
Central level	27.78 [20.07-35.48]	32.86 [26.85-38.86]	35.65	77.92

Regarding AFC, one OutpV cost US$8.41 and one InpBD cost US$36.66. Inpatient bed days for surgery cost up to US$101.72 compared to US$20.08 for non-surgery (Table 3). The share of user fees of the AFC differed among unit costs. On average, one OOP payment or TPP covered 49.1% of the AFC of outpatient visits and 55.2% of one inpatient bed day. The UF made up a higher proportion of the AFC of different unit costs at the central level compared to the provincial level, ranging between 56.8 and 77.9% and 44.6 and 61.3%, respectively. The highest share was for a non-surgical bed day at the central level (77.9%) and the lowest share for a surgical bed day at the provincial level (44.6%).

Impact implications:

In the estimation of the impact of sharing the unit costs of hospital services, one inpatient treatment episode of surgical treatment at either central or provincial hospital levels; and of a non-surgical treatment at central hospital level immediately made the near-poor households who are uninsured and had to pay OOP for the treatment at risk of CHE. Just one surgical inpatient treatment at central hospital level exceeded the 15% threshold of the household’s average annual income of the whole population (in 2008) (Table 4).

**Table 4:** User fees for surgical and non-surgical inpatient cases and their impact on users having to pay for the hospital service OOP

**Hospital service and level**	**UF per day (US$)**	**Average LOS (days)**	**UF for whole treatment episode (US$)**	**UF/annual income of near**-**poor household (%)**	**UF**/**average income per household in 2008 (%)**
**Surgical inpatient treatment case**					
**Province**	41.7	6.6	275.2	17.2	4.5
**Central**	102.5	8.7	891.7	55.8	15.1
**Non**-**surgical inpatient treatment case**					
**Province**	11.3	7.5	84.2	5.2	1.4
**Central**	27.7	10.4	288.1	18.1	4.8

## Discussion

Information about hospital unit costs are key requirements for many types of decision making, serving as input to assess the relative efficiency of treatment between hospitals, and are essential for budgeting and planning exercises [24]. The unit costs of inpatient bed days and outpatient visits are often available in high-income countries. Unfortunately, it is rare in Vietnam or in similar contexts in developing countries where the public hospital cost data are mostly nonexistent [37]. To fill this gap, using a large panel data of 4 consecutive years in 60% of general hospitals in Vietnam, the results of the current study reflect the real picture of Vietnam hospital health care services. The main result found was that generally up to 51% of outpatient visits and 45% of inpatient bed day costs are directly supported by the government, either through the state budget or through ownership and thus being responsible for depreciation. This indicates a higher proportion of hospital unit costs are covered by the government, compared to 30% of total health expenditure covered by public expenditure on health [12]. The main result in the current study was derived from a series of results on different hospital unit costs, which were found to be consistent with those of previous studies. The results of studies conducted by the Ministry of Health in 2006 (data for 2005) found the total cost per bed day (within 29 inpatient episodes) in provincial general hospitals to be 218,363VND (equal to US$36.8) [11]. Other studies by the Ministry of Health in 2005 (data from 2003 from 30 provincial hospitals) found that one inpatient bed day for surgery treatment (childbirth and appendicitis) cost 195,000VND (equal to US$33) and for internal treatment in the range of 94,000-340,000VND (US$15.8-57.4) [27]. At the central level, to our knowledge, there is only one study by Flessa & Dung from 2004 which gave results from Bachmai hospital, indicating that one outpatient visit costs US$0.86 and one inpatient bed day US$13.40 (those costs were converted to USD according to the exchange rate, and by PPP they were equal to US$2.3 for an outpatient visit and US$35.3 for an inpatient bed day), of which the inpatient bed day cost is consistent with our current results [13]. In comparison with other countries, our result is similar to the unit costs of secondary level hospitals in the much higher GDP per capita countries like Indonesia (cost of inpatient bed day: US$35.1), Equador (US$35.9), and Romania (US$39.0); and higher than those countries which have approximately the same GDP per capita, such as Algeria (cost of inpatient bed day: US$19.28) [37,1]. In comparison to the WHO categorized regions, our result is relatively lower than that of Western Pacific Region B (Vietnam belongs to this region) where an inpatient bed day costs US$63, and an outpatient visit costs US$34. It is similar to the Eastern Mediterranean Region D (Afghanistan, Pakistan, Iraq and Sudan, etc.) averages, though [38].

In consideration of DGS, for only one inpatient day of surgery at the central level the government has to subsidize up to US$78.07, that is as much as 10 outpatient visits at the provincial level. This support could be crucial for the poor or near-poor who have to pay for hospital services by OOP payment. However, the use of medical services (hospital admission) by the better off is 2.5-4.5 times greater than that of the poor [12]. The insured have almost twice the rate of admission than the uninsured [7], and insurance coverage was higher among those who have a higher ability to pay for health care [39]. That clearly implied an inequity in the benefits of hospital service utilization among different groups within the population. The richer could pay for the services but actually they gain greater benefit from the direct support of the government, which was originally targeted at the lower income group in the population [12,40,41].

*Policy implications*: The findings of the paper offer some suggestions for evidence-based policy solutions that will help decrease the prevalence of catastrophic health spending in Vietnam. One of three fundamental concerns of the government in health financing sources is to protect people from the financial consequences of ill health and having to pay for health services [42], aside from the poor who have been provided with free health insurance. The remaining near-poor households, will be subject to the negative impact of the FFS regime, that is, to be at risk of CHE. The government should shift from direct support to hospitals to the prepaid regime with free health insurance which would provide a larger proportion of the vulnerable group of low income people or households with the benefit of increased access to health care services.

The strength of our study is that the results relied on the panel data of quite a large number of hospitals (60% of the total number of general hospitals) in 4 consecutive years which allowed us to capture the outcome variation among hospitals caused by the differences of unobservable determinants and the correlation between differences of unobservable and observable determinants of behavior [35].

However, the limitations of this article are that, firstly, with the limited information on the health care system, poor quality of hospital statistics, and the multi-stage regressions used to estimate the unit costs, we were only able to relatively calculate some basic unit costs necessary for hospital policy considerations [6]. Secondly, the output measured here may provide a relatively poor fit, because the two groups of hospitals would have quite different total costs, while the total number of bed days, and also the number of outpatient visits, are the same [34]. Some of the cost may not be actually reflected such as the estimation of under table payment, the drugs out of hospital inventory which patient has to buy.

## Conclusions

While around 45% of hospital AFC is paid by DGS, the larger rest is covered by user fees. These have become a great financial burden for the uninsured near-poor households, as they have to pay for these out-of-pocket, which either leads to CHE and/or to an under-utilization of necessary services. If the rate of DGS were reduced, this would have the effect of increasing UF, but the savings to Government could be spent on subsidizing insurance to ensure that a larger part of the population can cover UF through insurance, especially the near-poor households, and thus to reduce their risk of CHE and/or under-utilization of services.

## Competing interests

The authors declare that they have no competing interest.

## Authors' contributions

DAV Initiating the idea for writing manuscript, Data collecting cleaning and analysis, drafting manuscript. SF Advising methodology. PM Involved in drafting manuscript. STH Data collection. KNL Involved in drafting manuscript, Advising methodology. RB The idea and direction of constructing manuscript, Correcting manuscript. All authors read and approved the final manuscript.
